# Engineered extracellular vesicles: potentials in cancer combination therapy

**DOI:** 10.1186/s12951-022-01330-y

**Published:** 2022-03-15

**Authors:** Jiangbin Chen, Qi Tan, Zimo Yang, Yang Jin

**Affiliations:** Department of Respiratory and Critical Care Medicine, NHC Key Laboratory of Pulmonary Diseases of Health Ministry, Union Hospital, Tongji Medical College, Huazhong University of Science and Technology, 1277 Jiefang Avenue, Wuhan, 430022 Hubei People’s Republic of China

**Keywords:** Extracellular vesicles (EVs), Engineered EVs, Cancer therapy, Combinational therapy

## Abstract

Extracellular vesicles (EVs) are a group of secretory vesicles with cell-derived membrane and contents. Due to the cargo delivery capability, EVs can be designed as drug delivery platforms for cancer therapy. Biocompatibility and immune compatibility endow EVs with unique advantages compared with other nanocarriers. With the development of this field, multiple ingenious modification methods have been developed to obtain engineered EVs with desired performance. Application of engineered EVs in cancer therapy has gradually shifted from monotherapy to combinational therapy to fight against heterogeneous cancer cells and complex tumor microenvironment. In addition, the strong plasticity and load capacity of engineered EV make it potential to achieve various combinations of cancer treatment methods. In this review, we summarize the existing schemes of cancer combination therapy realized by engineered EVs, highlight the mechanisms and representative examples of these schemes and provide guidance for the future application of engineered EVs to design more effective cancer combination treatment plans.

**Graphical Abstract:**

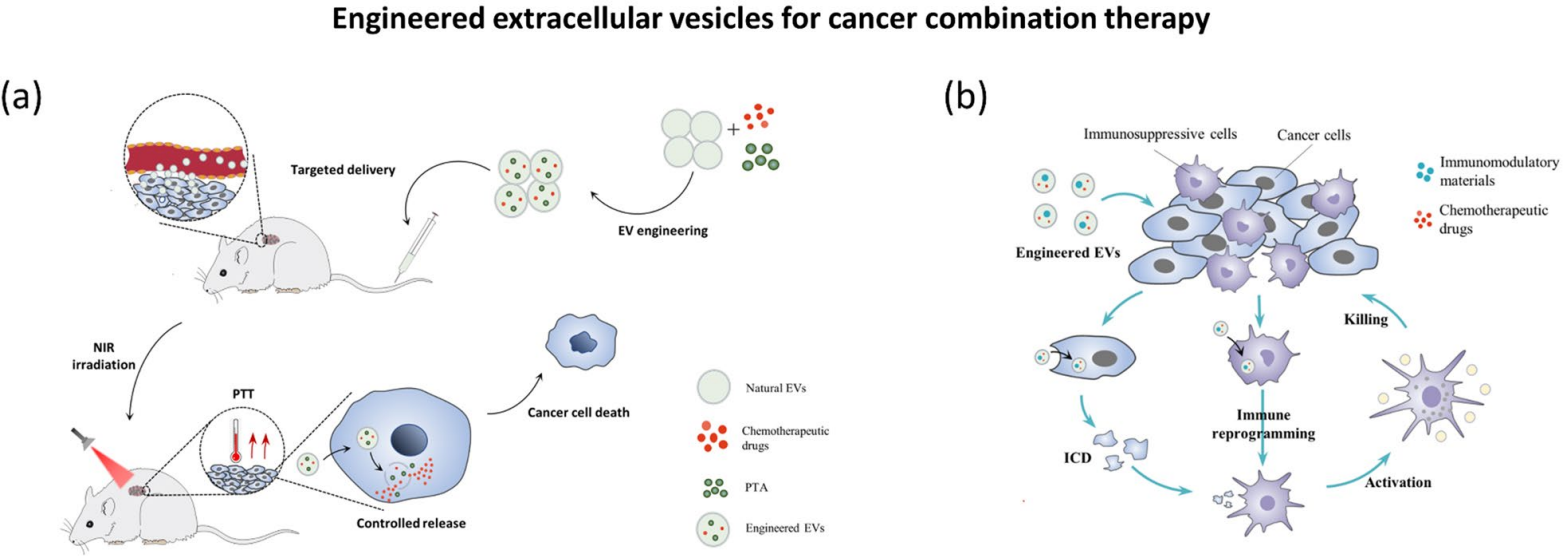

## Background

Extracellular vesicles (EVs) with cell-like structures originate from various cells [1]. It has been proved that they play essential roles in cell-to-cell communication under different physiological and pathological conditions [2, 3]. Based on the vesicle structure and the mechanism of intercellular communication, EVs are developed as drug delivery vectors and therapeutic agents for cancer therapy [4–6]. Compared with other nano-drug delivery platforms, natural EVs have better biocompatibility, prolonged blood circulation life span, lower immunogenicity and systemic toxicity. Moreover, features from parent cells endow EVs with unique targeting capability to their source cells [5, 7, 8]. However, natural EVs also have some unsatisfactory performance in cancer therapy. Up to now, no standardized protocol for isolation methods has been established. Also, large-scale manufacturing production and purification of EVs is still complicated [4]. The targeting ability of natural EVs would be weakened as trapped in some major organs such as liver or spleen [9, 10]. The anti-cancer immune response caused by natural EVs derived from tumor cells or immune cells is not strong enough [11, 12]. Low drug delivery efficiency, insufficient infiltration and penetration of natural EVs limit their anti-cancer efficiency [13]. To overcome the above-mentioned challenges of natural EVs, increasing studies are focusing on engineering EVs to obtain ideal anti-cancer effects. By various engineering methods such as loading functional cargoes and membrane modification, engineered EVs will show better performance and become more suitable in cancer therapy.

Combination therapy is a potential strategy to improve the therapeutic effect of cancer. Up to now, cancer is still a considerable threat to human health. Monotherapy is hard to fight against all kinds of carcinogenic factors in the complex tumor and tumor microenvironment (TME) [14]. Tumor heterogeneity determines the necessity of combination therapy. Recently, the research trend of cancer treatment has gradually removed from monotherapy to combination therapy. Combining two or more therapeutic approaches is expected to maximize the therapeutic efficacy of each method and minimize their disadvantages and side effects. For example, chemotherapy-involved combination therapy combats the multi-drug resistance (MDR) and endows chemotherapy drugs with higher performance in cancer treatments [15]. In addition, combination therapy with immune checkpoint blockade (ICB) can overcome the resistance and broaden the clinical application of ICB [16]. More and more clinical trials have shown that combinational therapy is more beneficial to patients than single therapy [17, 18]. Applying EVs in cancer treatment has been studied for decades. The researches range from the carrier property of natural EVs to the current use of engineered EVs that carry various anti-cancer substances and produce cancer vaccines. Recently, increasing studies are attempting to use engineered EVs to achieve cancer combination therapy. Engineered EVs with biocompatibility and targeting ability can be used for the targeted co-delivery of two and more anticancer materials. Their lipid bilayer membrane can also be modified to conduct combinational therapy. Based on these unique characteristics, engineered EVs have great potentials to be promising platforms for cancer combination therapy.

**Fig. 1:**
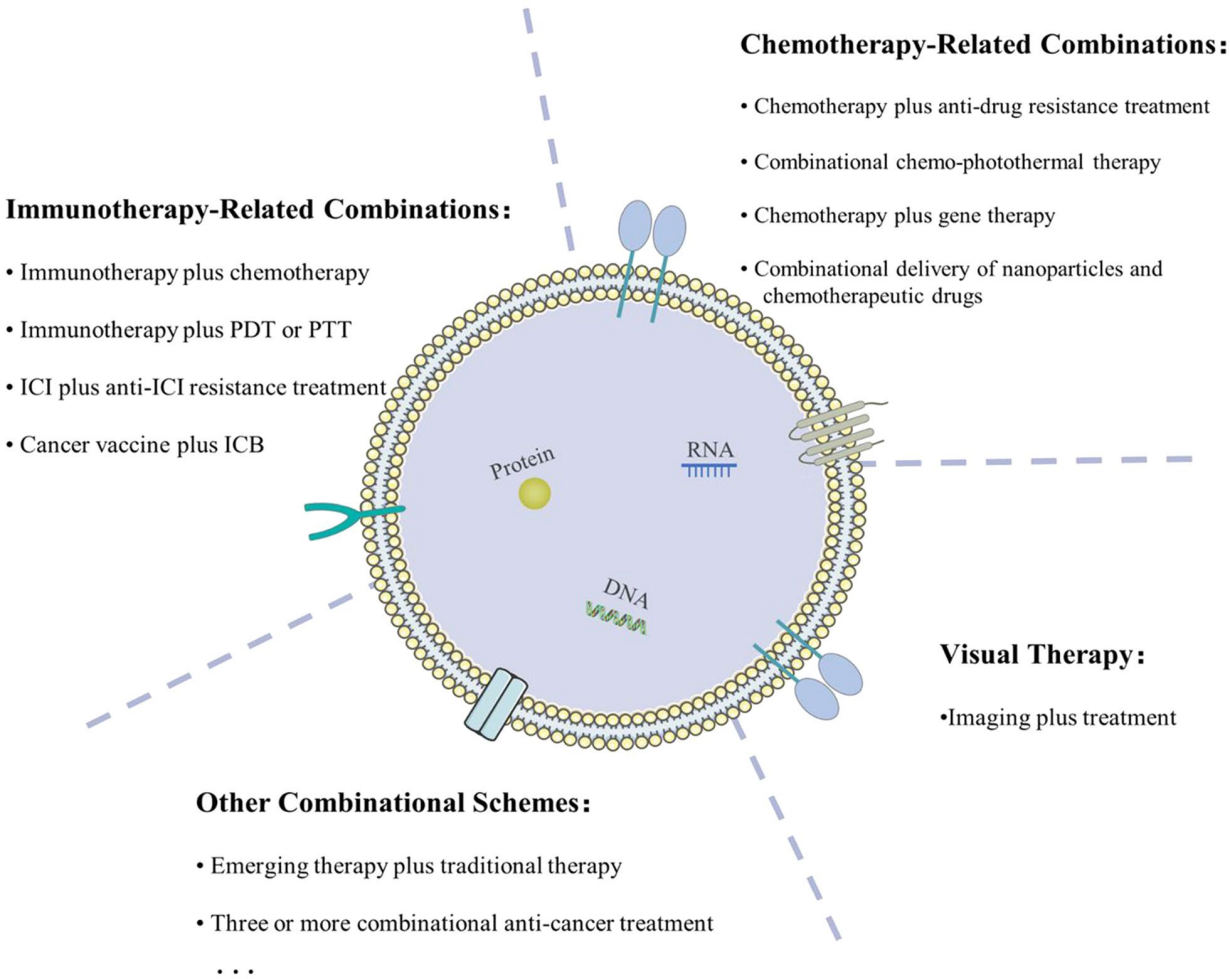
Existing schemes of cancer combination therapy based on engineered EVs. Chemotherapy-related combination, immunotherapy-related combination, and visual therapy are three main categories. Other schemes, such as the combination of new and traditional therapy, combination of three or more drugs, are increasingly mentioned in recent studies. *PDT* photodynamic therapy; *PTT* photothermal therapy; *ICI* immune checkpoint inhibitors; *ICB* immune checkpoint blockade

In this review, we begin by introducing the cell biology of EVs and the strategies of engineering EVs. Next, we summarize the existing schemes of cancer combination therapy based on engineered EVs (Fig. 1). We focus on understanding how these engineered EV-based combination schemes achieve better therapeutic effects in cancer, which can provide guidelines and references for further use of engineered EVs in cancer combination therapy and be helpful to deeply understand the future development of engineered EVs in cancer treatment.

## EV biology

According to sizes and origins, EVs can be classified into exosomes, microvesicles (i.e., microparticles), and apoptotic bodies. Exosome, whose size is from 40 to 200 nm, is generated when multivesicular bodies and plasma membranes fuse [19]. Microvesicles are particles ranging from 100–200 nm to 1 μm, generaing from membrane budding [20]. Apoptotic body is from the dying cell and has a diameter of 800 nm to 5 μm. Currently, apoptotic body is rarely used in cancer therapy due to its unsatisfactory size, so the knowledge of apoptotic bodies is not covered in this review.

**Fig. 2:**
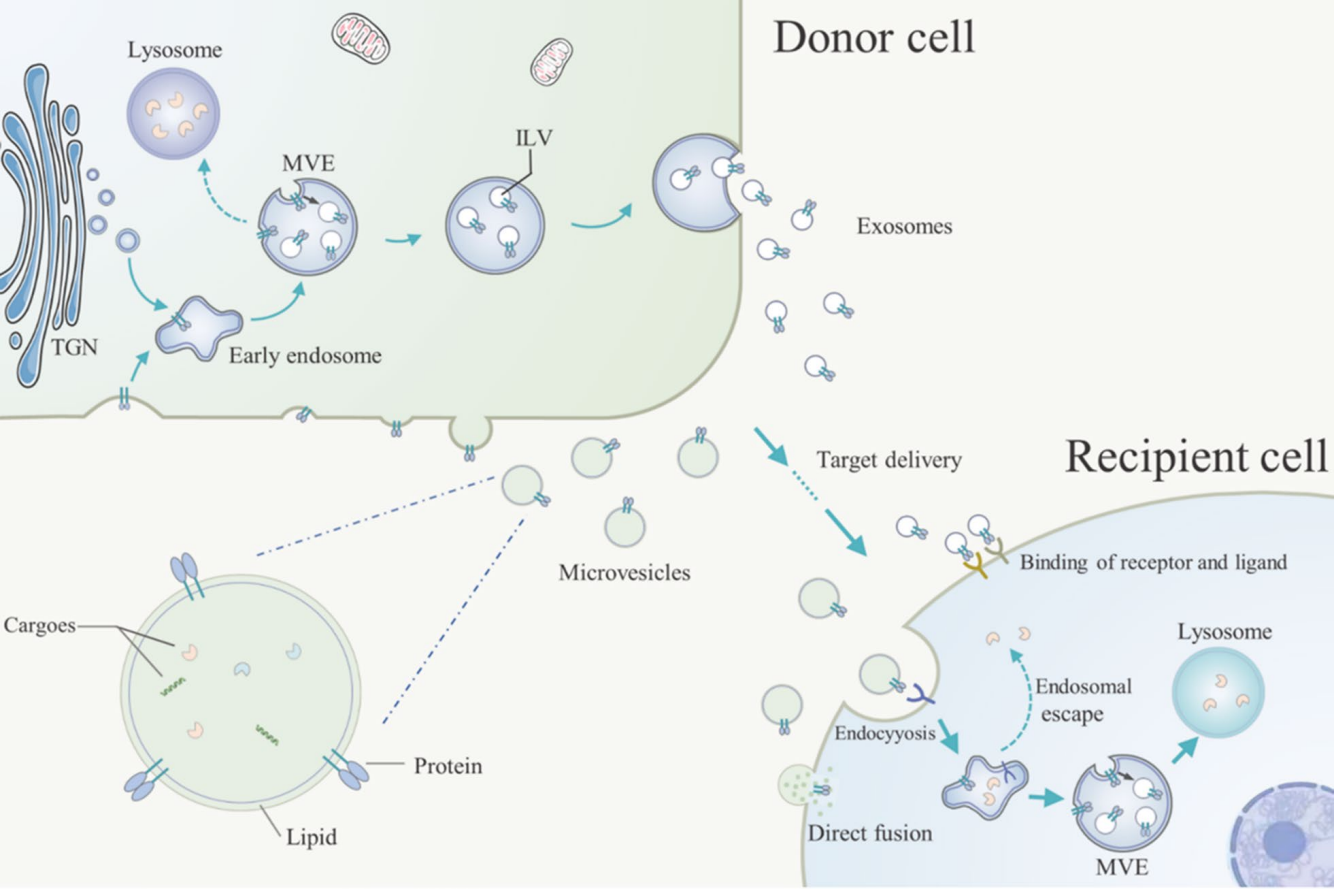
Brief biology of exosomes and microvesicles. Exosomes are formed in the endosome circulation as intraluminal vesicles (ILVs), which are derived from the multivesicular endosomes (MVEs). Microvesicles originate by a direct budding at the plasma membrane. After biogenesis, some exosomes and microvesicles will target the recipient cells. Through the binding of receptor and ligand, endocytosis pathway, or direct membrane fusion, exosomes and microvesicles will complete the cargo delivering and intracellular communication mission

Exosomes and microvesicles have different biogenetic mechanisms (Fig. 2). Exosomes are formed as intraluminal vesicles (ILVs) in the endosome circulation [21]. Vesicles derived from the endocytosis pathway or trans-Golgi network (TGN) form the early endosomes. During the following evolution process, early endosomes will develop into the multivesicular endosomes (MVEs) with part of endosomal membrane invagination into the lumen to form ILVs. Then some MVEs will fuse with the cell plasma membrane and release the ILVs into the extracellular environment in the form of exosomes [19, 22, 23]. As for microvesicles, understanding of their molecular mechanisms of biogenesis is still limited. It is known that microvesicles originate by direct budding at the plasma membrane [19, 23]. After biogenesis and release, EVs can enter the circulation and follow the blood flow to various positions in the body, completing the cargo delivering and intracellular communication mission. Due to the liposolubility, an essential feature of EVs is crossing a physical barrier such as the blood–brain barrier (BBB) [24]. It is one of Evs’ advantages as drug delivery system compared to other carriers [8]. Moreover, EVs are vesicles derived from autologous cells and regarded as “self” by the immune system, so they have good biocompatibility. Except for these features, EVs have also shown the capability for targeting recipient cells, which is closely related to the protein profile on EV surfaces. The targeting property is thought to be achieved by specific recognition and binding between the proteins at the EV surface and the ligands at the plasma membrane of the recipient cells [19]. Notably, the recipient cell can also be parent cell because the protein profile of EVs is similar to the producing cells, which is the reason to use tumor-derived EVs for cancer targeting therapy [12]. After EVs dock on the surface of the target cells, one possible outcome is that EVs activate the signaling pathway of recipient cells to generate corresponding responses through the binding of receptor and ligand. The other is that EVs release their cargoes into recipient cells through endocytosis or direct membrane fusion [19].

In conclusion, the EVs biology is deeply understood, making it possible to use engineered EVs for cancer therapy. Further exploration of the unknown points will provide more theoretical evcidence for developing better design and engineering of EVs for therapeutic applications.

## Strategies of EV engineering

**Fig. 3:**
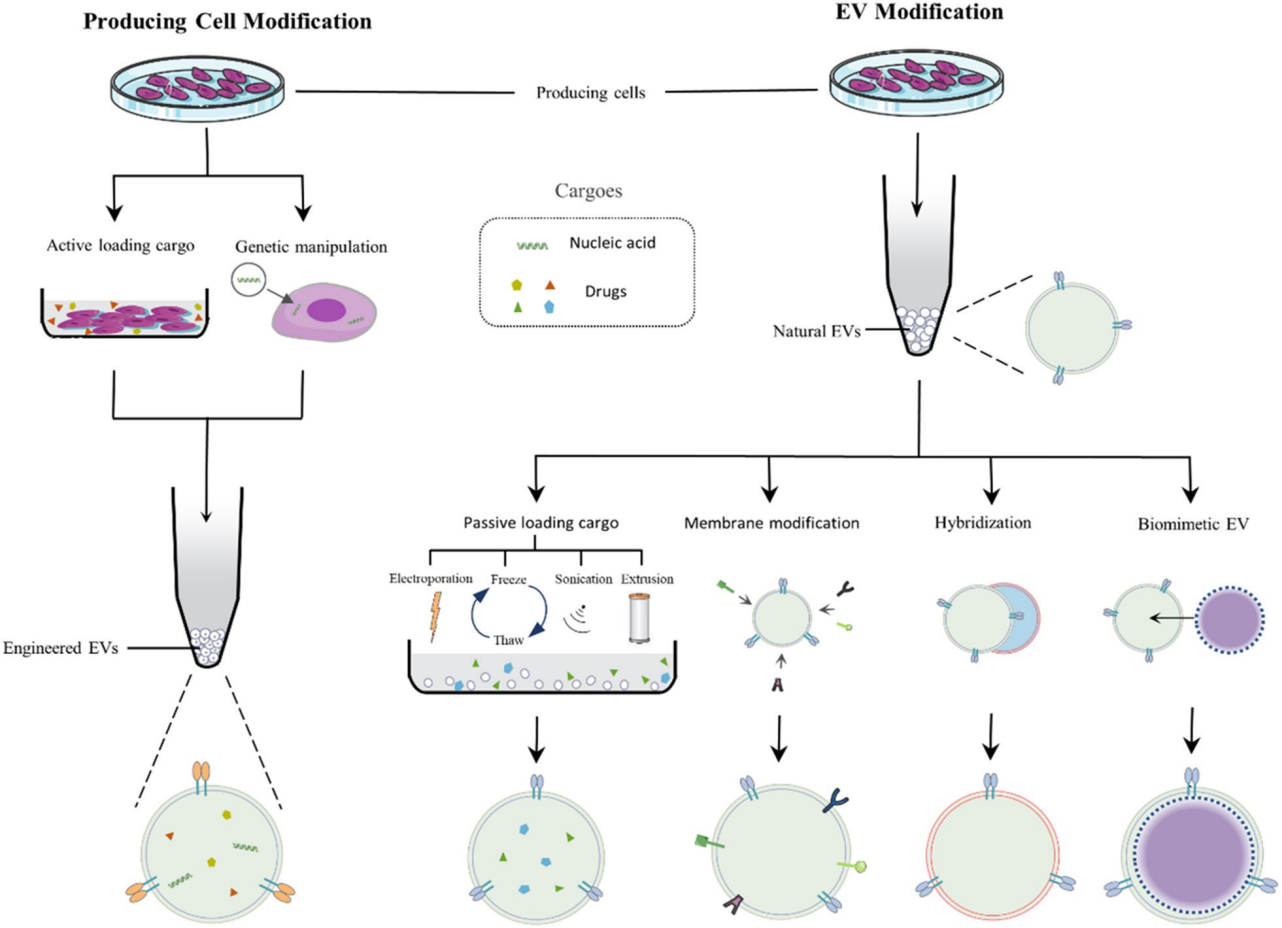
Strategies of EV engineering. EV modification (right) is a direct method to obtain engineered EVs, including passive loading cargo, EV membrane modification, hybridization, and biomimetic EV production. Producing cell modification (left) is the indirect method to get engineered EVs through engineering parent cells by active loading cargo or genetic manipulation

To broaden the application of EVs, various engineering strategies have been developed to improve EVs performance in cancer therapy [25] (Fig. 3). There is no best strategy and standard method to modify EVs. Each strategy has its own advantages and usage. Of note, the way of engineering EV can be based on diverse strategies. Combination therapy for cancer usually requires the simultaneous application of multiple engineering strategies.

### EV modification (direct method)

#### Passive loading cargo

Due to EVs’ poor active uptake ability, the loading quantity of simply mixing drug with EVs is usually unsatisfactory. Various passive loading methods have been developed to improve the cargo loading efficiency, such as electroporation [26], freeze and thaw cycles [27], sonication [28], and extrusion [29]. The principle of these physical methods is to increase the gap of the EV membrane or apply external force to make cargo enter the EVs. The point worthy to notice is that these passive methods may undermine EV membrane and affect EV integrity.

#### Modified EV membrane

The EV membrane structure is composed of lipids and proteins, which can be linked to some substances through chemical reactions to reduce clearance and enhance tumor-targeting capability. Click chemistry that utilizes copper-catalyzed azide-alkyne cyclo-addition reaction to attach specific molecules to EV membrane is one of the representatives [30–32]. Interlinkage between sulfhydryl and maleimide can also be employed to engineer EV membrane [33–35]. In these cases, chemical bonds contribute to the strong connection between target substances and EV membrane.

#### Hybridization

Hybrid vesicles can be designed by fusing EVs with other lipid nanovesicles like liposomes [36, 37]. The similar lipid membrane structure is beneficial to their fusion and the hybrid vesicles not only remain the characteristics of EVs but also obtain the advantages of other lipid nanovesicles.

#### Biomimetic EV production

Using individual biomimetic molecules such as EV membrane lipids and proteins to develop EV-based mimetics is feasible to produce better drug delivery platforms [38]. Encapsulating inorganic or organic nanoparticles with active EV membrane is a typical example of biomimetic EVs [39–41]. These mimetics have structure similar to natural EVs, which means they can perform excellent pharmacokinetics and biocompatibility.

### Producing cell modification (indirect method)

#### Active loading cargo

Majority of cells are able to perform active uptake of drugs in extracellular space. To obtain the EVs loaded with ideal cargo, co-incubation with producing cells is one of commonly used approaches. Materials with smaller sizes are more suitable for this strategy. Adjusting the conditions of co-incubation could influence the cargo loading efficiency and EV generation [42].

#### Genetic manipulation

Genetic manipulation through transfection tools like viral vectors to deliver cargo into producing cells is an effective approach for engineering parental cells, especially for loading nucleic acid [43, 44]. Donor cells that up-regulate nucleic acid expression can generate the target EVs with abundant nucleic acid. Future advances in genetic manipulation will further improve transfection efficiency to obtain ideal engineered EVs.

### Simultaneous modification of producing cell and EV

In some cases, target EVs for cancer combinational therapy requires multiple modifications to obtain more functions. Just engineering EVs or producing cells alone is hard to meet the design requirements. Combining both modified donor cells and EVs properly is regarded as a potential choice. For instance, Lv et al. prepared genetically engineered exosomes by transfecting parent cells. Then the exosomes were fused with thermosensitive liposomes and formed the hybrid nanoparticles. This simultaneous modification scheme successfully produced the target engineered EVs for cancer chemoimmunotherapy [45].

## Engineered EVs for chemotherapy-related combination therapy

Chemotherapy is a traditional method routinely used in cancer therapy for a long time. The outcomes of mono-chemotherapy are usually far from satisfactory in malignant tumors, so combinational strategies have been applied to obtain a higher performance [15]. Our previous research had used the microparticles derived from autologous tumors to achieve targeted chemotherapy in 2019 [46]. With the development of EVs, more and more studies focus on how to improve tumor-killing efficiency and reduce adverse reactions of chemotherapy through chemotherapy combinations with engineered EVs (Table 1).

**Table 1:** Engineered EVs for chemotherapy-related combination therapy

Scheme	Principle/mechanism	Source of EV	EV type	Engineering strategy	Cargoes	Membrane modification	Effects	Refs.
Chemotherapy combined with anti-drug resistance treatment	Co-delivery of P-gp siRNA and DOX by engineered EVs	RBC	Mimic vesicles	Incubated with vesicles	P-gp siRNA and DOX	Aptamer modification	Overcame drug resistance and killed MDR tumors	[51]
	Tumor cell-derived EVs can directly down-regulate P-gp expression	Bel7402 cells	Biomimetic EVs	Incubated with producing cells	PSINPs loaded with DOX		Possessed cellular uptake and cytotoxicity in both bulk cancer cells and cancer stem cells	[54]
		HEK293T cells	EVs	Incubated with producing cells, the obtained EV were mixed with LipHA	DOX	LipHA modification	Inhibited MDR tumor growth by 89% and extended animal survival time by approximately 50%	[55]
	Co-delivery of anti-miRNA and drugs	HCT-116^5FR^ cell line	Exosomes	Electroporation	MiR21i and 5-FU		Reversed drug resistance and enhanced the cytotoxicity in 5-FU-resistant colon cancer cells	[60]
		HEK293T cell culture	Exosomes	Cell transfection	Anti-miR-214		Reversed the resistance to cisplatin in gastric cancer	[61]
		4T1 cells	Tumor cell-derived EVs	Anti-miR-21 was transfected to producing cells, the obtained EVs and the GIONs were extruded through 100 nm porous membranes	Anti-miR-214 and GIONs		Attenuated DOX resistance, resulted in effective photothermal effect and demonstrated excellent T2 MR imaging	[62]
Combinational chemo-photothermal therapy	Co-delivery PTA and chemotherapy drugs. Appropriate temperature rise boosted the susceptibility of cancer cells to chemotherapy and reduce their drug resistance	DC2.4	EVs	Incubated with EVs	DOX	Self-grown gold nanoparticles	Improved cellular internalization, controlled drug release, enhanced antitumor efficacy and reduced side effects	[67]
		H22 cells	Microparticles	Electroporation	Bi_2_Se_3_ nanodots and DOX		Resulted in synergistic antitumor efficacy by combining PTT with chemotherapy	[68]
		HeLa cells	Microvesicles	Electroporation	ICG and DOX		Almost all the tumor cells could be killed by the synergistic effect of the released DOX and ICG	[69]
		4T1 cells	Biomimetic exosomes	Exosomes were mixed with MSNs and then processed through extrusion	ICG and DOX		ICG produced hyperthermia to collapse E-MSNs nanovehicles, achieving effective chemo-photothermal therapy	[70]
Chemotherapy combined with gene therapy	Exosomes as delivery platforms of CRISPR/Cas9	SKOV3 cells	Cancer-derived exosomes	Electroporation	CRISPR/Cas9-targeting PARP-1		Suppressed the expression of PARP-1 and enhanced the chemosensitivity to cisplatin, resulting in the apoptosisof cancer cells	[73]
	Co-delivery of therapeutic nucleic acids and chemotherapeutic drugs	Cal 27 cells	Microvesicles	Modified parent cells to get MVs with the membraneodified with biotin and folate, the Bcl-2 siRNA and PTX were packaged into these MVs by electroporation	Bcl-2 siRNA and PTX	Biotin and folate	Enhanced target and synergistic therapy toward breast cancer	[75]
Combinational delivery of nanoparticles and drugs	Modified chemotherapeutics-loaded NPs by using EV membrane	MDA-MB-231 cells	Biomimetic exosomes	The mixture of PCNPs and exosome membrane was coextruded by a 220 nm polycarbonate porous membrane	PTX-S-LA and CuB loaded PEG-PCL NPs		Enhanced breast cancer metastasis inhibition	[81]
		Patient’s urine	Biomimetic exosomes	Electroporation	PMA/Fe-HSA @DOX		Achieved superior synergistic low-dose chemo/chemodynamic performance g	[39]
		Macrophages	Biomimetic exosomes	Exosomes were mixed with PLGA and coextruded through a 100 nm membrane, then peptides were decorated on the exosomal membrane	Poly(lactic-co-glycolic acid) (PLGA) nanoparticles loaded DOX	Peptides that can target tumor cells	Exhibited tumor-targeting efficacy, inhibited tumor growth and induced intense tumor apoptosis	[82]

*P-gp* P-glycoprotein; *DOX* doxorubicin; *RBC* red blood cell; *MDR* multi-drug resistance; *PSINPs* porous silicon nanoparticles; *LipHA* lipid-grafted hyaluronic acid; *5-FU* 5-fluorouracil; *GIONs* gold–iron oxide nanoparticles; *PTA* photothermal agents; *PTT* photothermal therapy; *ICG* indocyanine green; *E-MSNs* exosome-camouflaged mesoporous silica nanoparticles; *PARP-1* poly (ADP-ribose) polymerase-1; *MVs* microvesicles, *NPs* nanoparticles; *PLGA* poly (lactic-co- glycolic acid)

### Engineered EVs for the combination of chemotherapy with anti-drug resistance treatment

MDR is the primary issue accounting for the failure of chemotherapy [47]. The biological background of MDR is very complicated, which contains inherent or acquired mechanisms of cancer cells [48]. Extensive studies have developed various strategies to overcome these MDR mechanisms, such as the usage of multidrug resistance inhibitors, RNA interference therapy and new anticancer drugs that can evade efflux reaction [49]. Engineered EVs as delivery platforms can realize co-delivery of these anti-MDR substances and chemotherapeutic drugs to suppress MDR and improve the chemotherapeutic effects.

**Fig. 4:**
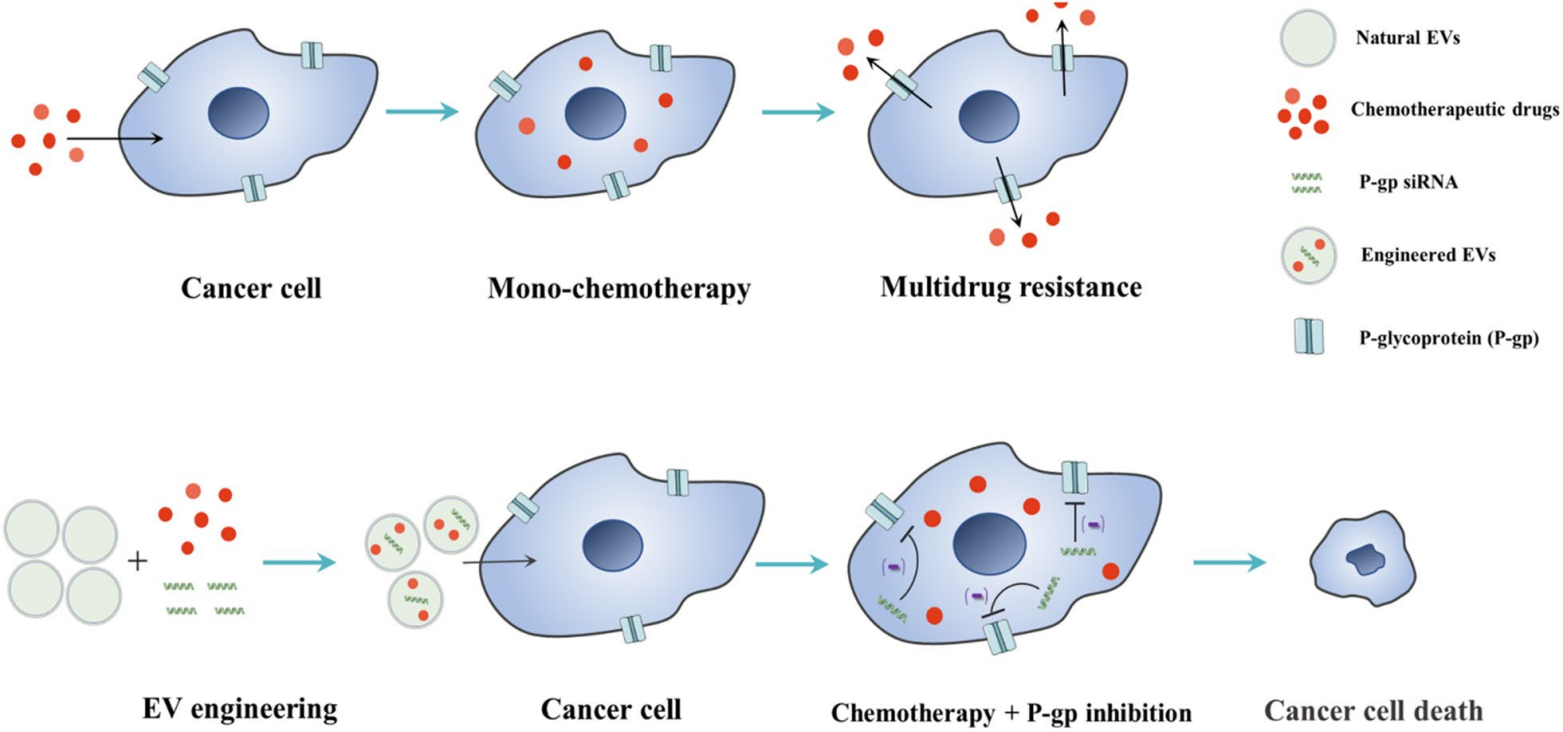
Engineered EVs for the combination of chemotherapy with anti-drug resistance treatment. Mono-chemotherapy often induces multi-drug resistance (MDR) in cancer cells. Engineered EVs as delivery platforms can realize co-delivery of anti-MDR substances like P-glycoprotein (P-gp) siRNA and chemotherapeutic drugs to suppress MDR and improve the chemotherapeutic effects

One of the most essential mechanisms of MDR is the overexpression of efflux transporters in tumor cell membrane, and P-glycoprotein (P-gp) is a prominent example of such drug efflux pumps [50]. Therefore, the combined delivery of P-gp siRNA and chemotherapeutic agents by engineered EVs is anticipated to enable the effective inhibition of drug-resistant tumors (Fig. 4). Wang et al. employed the alternative strategy of breaking and self-assembly to acquire the engineered mimic vesicles that replace the natural EVs. They achievied P-gp silencing combined chemotherapy strategy by loading P-gp siRNA and doxorubicin (DOX) into these mimic vesicles. The results demonstrated that these engineered drug carriers were able to overcome drug resistance and synergistically kill MDR tumors through P-gp silencing and DOX-induced growth inhibition [51]. Besides RNAi therapy, researchers found that tumor cell-derived EVs can directly down-regulate P-gp expression [52, 53]. Although the mechanism is not clear, this finding indicates that engineering tumor cell-derived EVs has the potential to overcome MDR and improve the chemotherapeutic effects. To realize that, Yong et al. developed biocompatible tumor exosome-based porous silicon nanoparticles loaded with DOX (DOX@E-PSiNPs) and showed DOX@E-PSiNOs could reduce the expression of P-gp and enhance cytotoxicity of DOX to cancer stem cells [54]. Liu et al. also used the functional EVs engineered with lipid-grafted hyaluronic acid (DOX@lipHA-hEVs) to reverse cancer drug resistance effectively [55]. All these studies showed that engineered EVs are potent tools to inhibit P-gp expression and enhance chemotherapeutic effect.

Dysregulated microRNAs (miRNAs) expression is another crucial pathological mechanism that contributes to the drug-resistance of cancer cells [56–59]. Transportation of antisense inhibitors against such miRNAs is a potential strategy for anti-drug resistance treatment. As mentioned above, EVs are promising platforms for RNA transportation. Therefore, researchers develop engineered EVs to deliver anti-miRNA and chemotherapeutic drugs to improve the effect of chemotherapy. For example, Liang et al. exploited engineered exosomes loaded with 5-Fluorouracil (5-FU) and miRNA-21inhibitor (miR-21i) via electroporation, successfully overcoming drug resistance in colon cancer cells and enhancing the cytotoxicity of 5-FU [60]. Wang et al. showed that exosomes carrying anti-miR214 could effectively reverse chemoresistance to cisplatin in gastric cancer [61]. Rajendran et al. demonstrated that anti-miR-21 delivery mediated by tumor cell-derived EVs could attenuate DOX resistance in breast cancer cells with a three-fold higher cell kill efficiency than in cells treated with DOX alone [62]. These prominent results suggest that delivery of anti-miRNA and chemotherapeutics based on engineered EVs is feasible to reverse drug resistance and improve chemotherapy performance.

### Engineered EVs for combinational chemo-photothermal therapy

Photothermal therapy (PTT) is an emerging method that applies photothermal agents (PTAs) to convert optical energy into heat to kill cancer cells [63]. As the temperature rises, most chemotherapy drugs will become more effective. Hyperthermic intraperitoneal chemotherapy is one of the most typical examples [64]. In addition, appropriate temperature rise can boost the susceptibility of cancer cells to chemotherapy and reduce their drug resistance [65, 66]. Therefore, combining chemotherapy and PTT can improve the synergistic efficacy of comprehensive therapy and reduce the dose of chemotherapeutic drugs to minimize the side effect.

**Fig. 5:**
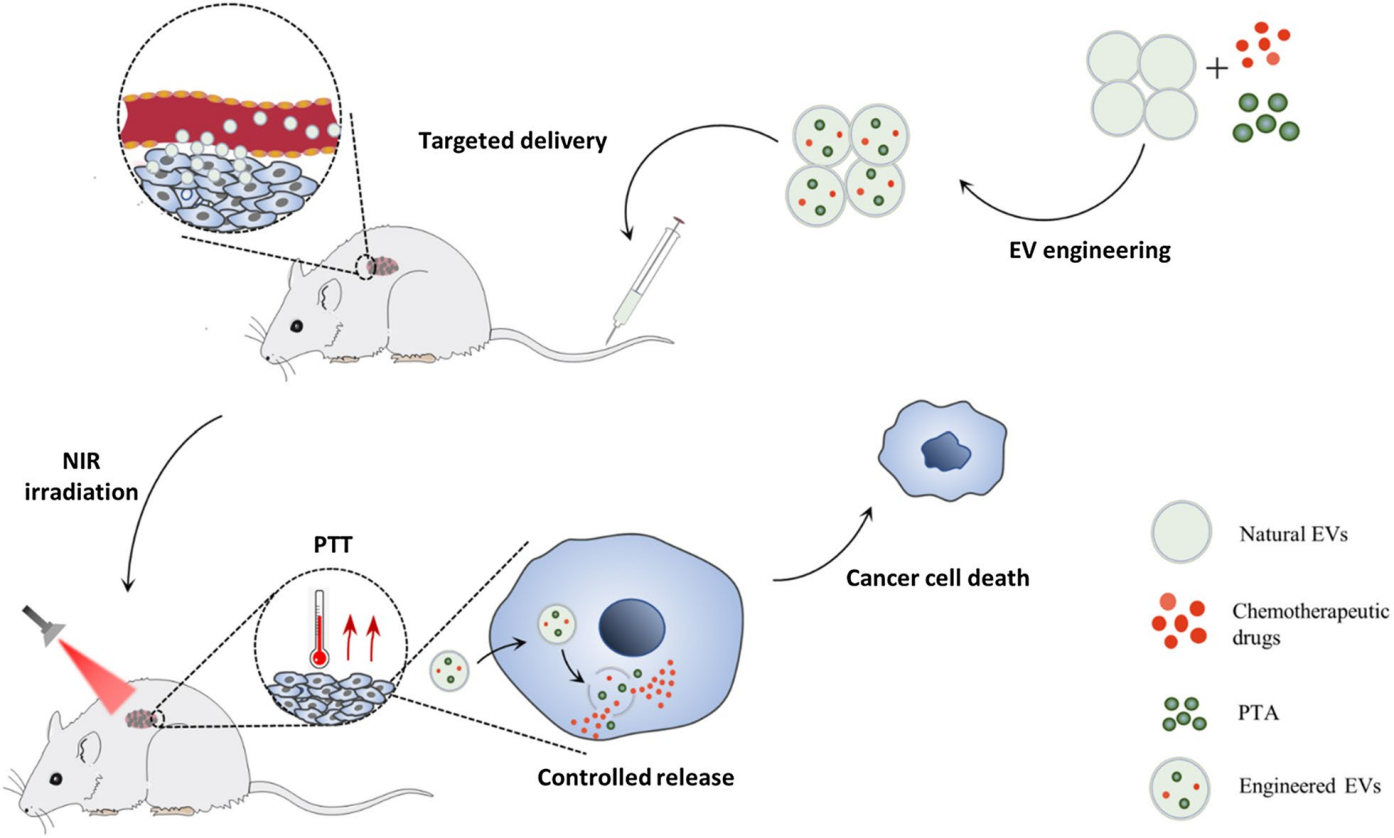
Engineered EVs for combinational chemo-photothermal therapy. Engineered EVs can co-delivery chemotherapeutic drugs and photothermal agents (PTAs) to tumor sites. Under near-infrared (NIR) irradiation, PTAs convert optical energy into heat for photothermal therapy (PTT). The elevated temperature can induce the collapse of the structure of engineered EVs and the controllable release of chemotherapeutic drugs to realize chemotherapy. Chemotherapy combined with PTT induces cancer cell death

As novel vehicles, engineered EVs can co-delivery chemotherapeutic drugs and PTAs (Fig. 5). The targeting capability and enhanced internalization of engineered EVs will further decrease the dose of chemotherapeutic drugs without limiting efficacy, avoid the distribution of PTA to normal tissues and cause fewer adverse effects. Engineered EVs significantly improve the feasibility and safety of combinational chemo-photothermal therapy. Zhang et al. had achieved this combinational chemo-photothermal strategy through generating an EVs-based self-grown gold nanostructure encapsulated with DOX (EVdox@AuNP). They proved that the popcorn-like EVdox@AuNP was capable of completing photothermal transduction and release DOX for chemotherapy, enhancing antitumor efficacy with tumor inhibitory rate up to 98.6% and reducing side effects [67]. Wang et al. also developed an engineered cell-derived microparticle for synergistic photothermal/lose-dose chemotherapy. To get higher load efficiency without disrupting the membrane integrity of EVs, they proposed an alternative approach that the parent cells were firstly engineered with transfer PTT agent Bi_2_Se_3_ and DOX, then irradiated with UV light to produce the ideal microparticles (Bi_2_Se_3_/DOX@MPs). Their results indicated that injection of Bi_2_Se_3_/DOX@MPs into tumor-bearing mice resulted in remarkably synergistic antitumor efficacy under 808 nm laser irradiation by combining PTT with low-dose chemotherapy [68].

Highly controlled release is another outstanding advantage of applying engineered EVs for combinational chemo-photothermal therapy. In normal conditions, chemotherapeutic drugs and photothermal materials are packaged in the membrane of engineered EVs. After the target area is irradiated, the heat generated by the PTT will increase the local temperature rapidly. The elevated temperature can induce the collapse of the structure of engineered EVs and the controllable release of therapeutic agents to realize precise cancer therapy. Zhu et al. utilized the electroporation technique to establish such a biocompatible delivery system. They prepared the microvesicles (MVs) loaded with DOX and photosensitizer indocyanine green (ICG). They showed that the ambient temperature increased by external near-infrared (NIR) laser irradiation induced the crack of MVs and the controllable release of DOX and ICG. In this case, tumor cells would be killed by the synergistic effect of chemotherapy and PTT [69]. Tian et al. also developed a controllable delivery system for co-loading DOX and ICG. In their research, tumor-derived exosomes were mixed with mesoporous silica nanoparticles (MSNs) loaded with DOX and ICG then exosome-camouflaged MSNs (ID@E-MSNs) was constructed through extrusion. Likewise, their outcomes demonstrated that ID@E-MSNs could produce hyperthermia to collapse nano-vehicles and accelerate drug release, achieving effective chemo-photothermal therapy under 808 nm NIR irradiation [70]. In short, all the researches above indicate that engineered EVs as delivery platforms perform strong load capacity and good biocompatibility, which means they have great potential in combinational chemo-photothermal therapy.

### Chemotherapy combined with gene therapy based on engineered EVs

Gene therapy is a promising approach for delivering therapeutic nucleic acids into cells to correct or modify genetic information [71]. Genetic mutation is the primary cause of cancer. Some mutated genes have been proved to be able to affect cancer therapeutic effect. Using gene therapy to target these mutated genes at the genetic level can fundamentally improve the effectiveness of cancer treatment, including chemotherapy. As an ideal nano-delivery platform, engineered EVs are capable of co-delivering of therapeutic nucleic acids and chemotherapeutic drugs. Moreover, the targeted transport by engineered EVs can reduce chemotoxicity and genetic contamination to other normal cells while achieving cancer combination therapy.

The clustered regularly interspersed short palindromic repeats (CRISPR) and CRISPR-associated proteins 9 (Cas9) system is a powerful tool for genomic editing [72]. Kim et al. explored the cancer-derived exosome as an intracellular delivery system for CRISPR/Cas9. They used the CRISPR/Cas9-loaded exosomes to suppress the expression of poly (ADP-ribose) polymerase-1 (PARP-1), which was closely related to the repair of chemotherapy-induced DNA damage in cancer cells. They showed that the inhibition of PARP-1 by this engineered delivery system with genome editing tool enabled synergistic cytotoxicity effect on ovarian cancer cells when combined with platinum-based chemotherapy [73].

Oncogene B-cell lymphoma-2 (Bcl-2) is involved in the control of cell anti-apoptotic defense mechanism to resist anti-cancer therapy [74]. To inhibit Bcl-2 expression and potentiate chemotherapy, Zhu et al. established the folate-engineered microvesicles for packaging Bcl-2 siRNA and paclitaxel (BFMVs@Bcl-2 siRNA@Paclitaxel). Compared with single model therapy, this MV-based drug delivery system performed high tumor-targeting capability, down-regulated Bcl-2 expression level, and significantly improved the synergistic antitumor efficacy of chemotherapy and gene therapy [75].

### Engineered EVs for the combinational delivery of nanoparticles and chemotherapeutic drugs

Nanoparticles (NPs) are widely used in drug delivery due to their unique physiological properties [76]. Smaller size and larger specific surface area provide NPs with high efficient loading capacity [77]. In the TME, NPs can prevent the degradation of drugs from the acidic and proteolytic environment [78]. More importantly, NPs show increased accumulation in the tumor site through the enhanced permeability and retention (EPR) effect [77, 79, 80]. Therefore, combining nanoparticles with cancer chemotherapy is an ideal strategy in theory. However, some obstacles limit the application of NPs in chemotherapy, including insufficient targeting property, potential toxicity, and high clearance in body. Engineered EVs may be a possible solution to these limitations of NPs. Studies have developed a strategy that modifies NPs by using EV membrane to assist NPs in evading immune clearance and targeting tumor cells. Such engineered biomimetic EVs can realize high loading efficiency, low toxicity, long circulation time, effective targeting, and enhanced accumulation for better chemotherapy.

Many studies have made breakthrough progress in this area. For example, Wang et al. packaged the reactive oxygen species (ROS)-responsive thioether-linked paclitaxel-linoleic acid conjugates (PTX-S-LA) and cucurbitacin B (CuB) into nano-sized polymeric micelles and further used exosome membrane to decorate these nanoparticles to obtain a sequential-bioactivating prodrug nanoplatform (EMPCs) [81]. They proved that EMPCs exhibited amplified prodrug bioactivation, prolonged blood circulation, selective targeting of tumor cells, and enhanced tumor penetration. Pan et al. also developed a biomimetic nano-vector Exo-poly (isobutylene-alt-maleic anhydride) /Fe_3_O_4_-human serum albumin (HSA)@DOX with urinary exosome membrane. It could modify Fe_3_O_4_ nanoparticle, DOX, and HSA as a chemo-chemodynamic nano-platform for targeted homologous prostate cancer therapy [39]. To further improve the targeting ability, Li et al. developed macrophage-derived exosomes-coated poly (lactic-co-glycolic acid) nanoplatforms and further modified them with peptides that can target tumor cells. Their study revealed that the engineered exosomes-coated nanoparticle not only significantly prolonged the circulation time of DOX, but also achieved higher tumor targetability and better accumulation in tumor tissues during triple-negative breast cancer chemotherapy [82]. Collectively, all these studies show that applying engineered EVs for combinational delivery of nanoparticles and chemotherapeutic drugs is a feasible approach to combine the advantages of nanoparticles and EVs for high-efficiency chemotherapy.

## Engineered EVs for immunotherapy-related cancer combination therapy

Cancer immunotherapy that utilizes immune system to combat tumors is a validated and critical approach for cancer treatment. The significant clinical benefits achieved by immunotherapy have revolutionized the treatment of many advanced cancers. However, low response rates and immune-related adverse events limit the broad application of cancer immunotherapy. Adopting combinational strategy is a potential way to overcome these limitations [83, 84]. Applications of engineered EV in this field have made some remarkable breakthroughs to realize immunotherapy-related combination therapy (Table 2).

**Table 2:** Engineered EVs for immunotherapy-related cancer combination therapy

Scheme	Principle/mechanism	Source of EV	EV type	Engineering strategy	Cargoes	Membrane modification	Effects	Refs.
Immunotherapy combined with chemotherapy	Co-delivery of chemotherapeutic drugs and immunomodulator. Chemotherapy induce ICD and activate immune effector cells	Fibroblasts	Thermosensitive exosome—liposome hybrid nanoparticle	Fusion of engineered exosomes and drug-loaded thermosensitive liposomes by freeze—thaw method	GM-CSF and docetaxel	CD47 overexpression	Inhibited tumor development	[45]
		BM-MSCs	Exosomes	Electroporation was applied to load galectin-9 siRNA, OXA-MAL was added to the exosomes via vortexing	Galectin-9 siRNA	OXA	Elicited anti-tumor immunity and inhibited tumor growth	[96]
Immunotherapy combined with PDT or PTT	Co-delivery of immunostimulatory and PS or PTA. PDT and PTT can induce ICD and covert “cold” tumor into “hot” tumor	MSCs	EVs	Turbulence induced high-yield production of MSC-derived EVs encapsulating mTHPC	Photosensitizer mTHPC		Permitted important tumoral necrosis and decreased intratumor proliferation	[99]
		CT26 cells	Hybrid vesicles	Fusion of exosomes and thermosensitive liposomes	ICG and adjuvant R837	CD47 overexpression	Eliminated the tumors	[100]
ICI combined with anti-ICI resistance treatment	Anti-ICI resistance treatment could improve ICI resistance	RAW264.7 macrophages	Microparticles	RAW264.7 macrophages were incubated with DSPE-PEG-Man and then were treated with metformin	Metformin	Mannose	Improved anticancer efficacy and long-term memory immunity	[104]
Therapeutic cancer vaccine combined with immune checkpoint blockade	Engineered EVs as vaccines, ICI can boost and maintain the vaccine’s effect to produce persistent responses	B16F10 cells/CT26 cells	Tumor-derived microparticles (T-MPs)	Incubated nano-Fe3O4 with producing cells, the obtained MPs were incubated with CpG-loaded liposome	Nano-Fe_3_O_4_	CpG-loaded liposome	Inhibited tumor progression	[34]
		BMDCs	Exosomes	Exosomes prepared from ovalbumin-pulsed, activated DCs were modified with anti-CTLA-4 antibody		Anti-CTLA-4 antibody	Slowed down tumor growth	[114]

*GM-CSF* granulocyte–macrophage colony-stimulating factor; *OXA* oxaliplatin; *MAL* N-(2-Aminoethyl) maleimide; *PDT* photodynamic therapy; *PTT* photothermal therapy; *PS* photosensitizers; *PTA* photothermal agents; *ICD* immunogenic cell death; *ICG* indocyanine green; *mTHPC* meta(tetrahydroxyphenyl)-chlorin; *ICI* immune checkpoint inhibitors

Notably, immune cell-derived EVs play critical roles in this field. Recent studies have shown that immune cell-derived EVs can facilitate the immune response process by mediating crosstalk between different cells in the cancer-immunity cycle [11, 85, 86]. Applying immune cell-derived EVs can regulate the state of target immune cells to promote anti-cancer immunity. For example, Choo et al. showed that exosome-mimetic nanovesicles derived from M1 macrophages could repolarize M2 tumor-associated macrophages (TAMs) to M1 macrophages and induce antitumor immune responses in vitro and in vivo [87]. Matsumoto et al. proved that the small EVs originating from mature dendritic cells (DCs) could repolarize macrophages and activate DCs for tumor antigen-based cancer immunotherapy [88]. In addition, compared with parent cells, immune cell-derived EVs have some unique features. Their membrane area is smaller. Thus, they have higher density of functional signaling molecules. Their nano-scale particle size and non-cellular activity make them penetrate tumors easily, retent longer, and less susceptible to the tumor immunosuppressive microenvironment [89, 90]. These features make immune cell-derived EVs more potent in immunotherapy-related cancer treatment. Take T cell as an example, Fu et al. proved that EVs released by T cells expressing chimeric antigen receptor (CAR) are relatively safe and effective compared with CAR-T therapy for solid tumors. CAR exosomes have the potential to overcome the flaws of CAR-T cells to accumulate and replicate in TME [91]. Despite these advantages of immune cell-derived EVs, we should also pay attention to the duality of their effects on the immune system. That is, they may exhibit both immune-stimulatory and immunosuppressive roles. The specific effect of immune cell-derived EVs is closely related to the state of parent cells [4, 11]. Therefore, adjusting parent cells to an appropriate status is important when applying immune cell-derived EVs to immunotherapy-related cancer treatment.

### Engineered EVs for the combination of immunotherapy with chemotherapy

**Fig. 6:**
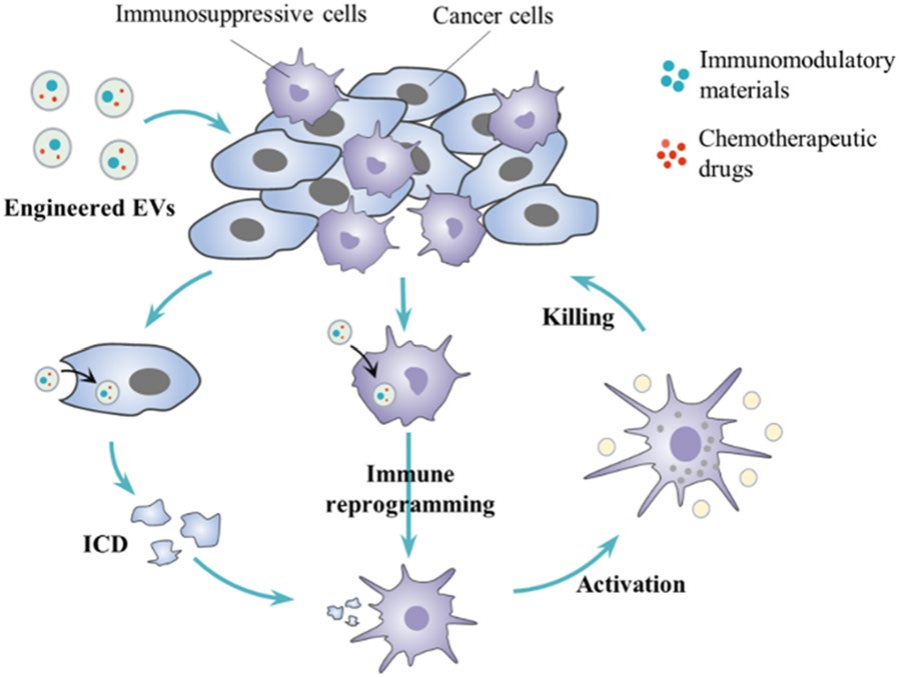
Engineered EVs for the combination of immunotherapy with chemotherapy. Immunomodulatory materials and chemotherapeutic drugs are co-delivered to the tumor microenvironment by engineered EVs. Immunomodulatory materials reprogram immunosuppressive cells to immune effector cells. Chemotherapy can induce immunogenic cell death (ICD) and expose more tumor antigens, facilitating better immunotherapy

Immunotherapy functions mainly through immune effector cells. In the TME, immune effector cells lose anti-tumor abilities and changed to immunosuppressive phenotype due to complex mechanisms [92]. For instance, M1 macrophages inhibit tumor growth while M2 macrophages in the TME, such as TAMs, suppress anti-cancer immunity and promote tumor growth [93]. Therefore, rewiring immunosuppressive cells to generate anti-cancer immunity is essential for immunotherapy. Studies demonstrate that chemotherapeutic agents can produce immunostimulatory functions to reactivate immune effector cells [94]. Meanwhile, chemotherapy can induce immunogenic cell death (ICD) then expose more tumor antigens, facilitating better immunotherapy [95]. Given these functions of chemotherapy, combining immunotherapy with chemotherapy to increase the synergistic anti-tumor effects of monotherapy is reasonable. Engineered EVs act as drug delivery platforms can be used for co-delivering immunomodulatory materials and chemotherapeutic drugs (Fig. 6). Enhanced tumor site accumulation and endocytosis phagocytosis effects of engineered EVs are beneficial to transport immunomodulatory drugs into immunosuppressive cells to produce better immune reprogramming effects.

For instance, to improve the treatment of metastatic peritoneal carcinoma (mPC) by chemoimmunotherapy, Lv et al. designed genetically engineered exosomes-thermosensitive liposomes hybrid nanoparticles (gETL NPs) for targeted delivery of granulocyte–macrophage colony-stimulating factor (GM-CSF) and docetaxel (DTX). They prepared genetically engineered exosomes through transducing lentiviral vectors into fibroblasts for overexpression of CD47 and GM-CSF. CD47 mediated phagocytosis enhancement and GM-CSF promoted repolarization of macrophages towards M1 profile. Then G/D-gETL NPs were generated by the fusion of these engineered exosomes and thermosensitive DTX-loaded liposomes. They showed that G/D-gETL NPs effectively repolarized the M2 phenotype macrophages to M1 phenotype and inhibited tumor development in mice through combining immunotherapy and chemotherapy [45]. In another research, to enhance immunotherapy and reprogram tumor microenvironment in pancreatic ductal adenocarcinoma (PDAC), Zhou et al. constructed an exosome-based delivery system (iEXO-OXA) that loaded with galectin-9 siRNA and surficially modified with oxaliplatin (OXA) to reverse immunosuppression and trigger ICD. They demonstrated that iEXO-OXA improved tumor targeting efficacy and increased drug accumulation in the tumor site. OXA-mediated cytotoxicity promoted tumor cells death and induced ICD. Galectin-9 siRNA-mediated immunotherapy elicited anti-tumor immunity through tumor-suppressive macrophage polarization, cytotoxic T lymphocytes recruitment, and regulatory T cells downregulation. IEXO-OXA, combining immunotherapy and chemotherapy, achieved significant therapeutic efficacy in orthotopic PDAC mice [96]. Both studies proved that immunotherapy combined with chemotherapy based on engineered EVs could broaden the application of immunotherapy and produce stronger anti-cancer immunity.

### Engineered EVs for the combination of immunotherapy with photodynamic therapy or photothermal therapy

Similar to chemotherapy, photodynamic therapy (PDT) and PTT can induce ICD and stimulate adaptive immune responses, which will cause long-term immunological memory. Converting immunologically “cold” into “hot” tumor by PDT or PTT could effectively increase the sensitivity of immunotherapy [97, 98]. However, PDT or PTT application in cancer treatment is limited by the low tumor selectivity and significant adverse effects of photosensitizers (PS) or PTA. To overcome these obstacles, engineered EVs with superior delivery functions are explored to deliver PS or PTA. The targeted capability of engineered EVs can reduce the damage of these foreign materials to normal cells and achieve precise PDT or PTT. At the same time, the immunostimulatory effects of PDT or PTT will promote immunotherapy and achieve a combined therapeutic effect on cancer.

In the Pinto et al. study, the PS meta(tetrahydroxyphenyl)-chlorin (mTHPC) was encapsulated into EVs derived from mesenchymal stem/stromal cells (MSCs) to form the fourth generation of immune active PS vectors (EVs-mTHPC). Turbulence, as a pioneering method, was developed for large-scale production of EVs-mTHPC that was compatible with requirements of clinical translation while preserving the topology and integrity of natural EVs. The results of this study showed that high yield EVs-mTHPC performed higher tumor selectivity, induced tumor necrosis, promoted antitumor immune cell infiltration, and finally achieved immune reprogramming precision photodynamic therapy in carcinomatosis [99]. In another typical study conducted by Cheng et al. hybrid therapeutic nanovesicles (I/R@hGLV) were generated by fusing the CD47-overexpressed exosomes with thermosensitive liposomes loaded with photothermal agent ICG and immune adjuvant R837. The overexpression of CD47 on the engineered exosomes was in favor of avoiding the immune clearance by the mononuclear phagocyte system and prolonging the blood circulation time. Moreover, the CD47-expression could competitively bind with SIRPα, prior to tumor cells, leading to enhancing tumor cell phagocytosis by macrophages. The cargo ICG could achieve better PTT and induce ICD, triggering strong immune responses with the help of adjuvant R837. In tumor-bearing mice, I/R@hGLV efficiently inhibited tumors by combining cancer immunotherapy and PTT [100]. These two studies showed that immunotherapy combined with PDT or PTT based on engineered EVs is a feasible approach to obtain more potent anti-tumor effects.

### Engineered EVs for the combination of immune checkpoint inhibitor (ICI) with anti-ICI resistance treatment

Immune checkpoint inhibitors (ICI) play key roles in cancer immunotherapy and the significant clinical effects achieved by ICI have greatly improved the outcome of many advanced cancers. However, despite the success of ICI, resistance to these agents restricts the production of immune responses. Mechanisms of the ICI resistance are complex, which include lack of sufficient T cell infiltration and tumor immunosuppressive microenvironment [101]. Anti-ICI resistance treatment is required to increase the potential response rates. Repolarizing TAMs is one of the promising strategies to improve ICI resistance by enhancing T-cell antitumor immunity and ameliorating tumor immunosuppression [102, 103]. As mentioned earlier, engineered EVs are potent tools to reset M2-like TAMs to M1 phenotype. In the Choo et al. research, exosome-mimetic nanovesicles derived from M1 macrophages (M1NVs) were used to reset M2 TAMs to M1 macrophages, which showed the potential anti-cancer efficacy of ICI [87]. To better target and repolarize TAMs, Wei et al. further engineered macrophage-derived microparticles through mannose modification and loading metformin (Met@Man-MPs). In Met@Man-MPs, mannose could target CD206/MRC1, the highly expressed receptor of M2 TAM, and the cargo metformin contributed to repolarizing TAMs to M1 profile through the AMPK-NF-κB signaling pathway. They proved that Met@Man-MPs could degrade tumor collagen to support the infiltration of CD8 + T cells into tumor interiors and enhance tumor penetration of ICI, boosting immune checkpoint inhibition therapy and improving anticancer efficacy and long-term memory immunity after combinational treatment [104]. Although the number of such research is limited, the available data have shown that engineered EVs are suitable platforms for combining ICI with anti-ICI resistance treatment to increase the response rates of immune checkpoint blockade. Future development of engineered EVs relevant to anti-ICI resistance methods combined with ICI will enrich this combinational scheme.

### Engineered EVs for the combination of therapeutic cancer vaccine with immune checkpoint blockade

Therapeutic cancer vaccine has emerged as a powerful therapy strategy by stimulating adaptive immunity against specific tumor antigens to control over tumor growth and induce regression of tumors [105]. Cancer vaccines usually comprise four key components: tumor antigens, formulations, immune adjuvants, and delivery vehicles [106]. Recently, EVs derived from tumor cells or immune cells have been engineered to cover these four components, which has become an attractive method to develop cancer vaccines. Tumor-derived EVs retaining multiple tumor antigens, coupled with their own delivery function, only need to be modified with adjuvants before they used as suitable tumor vaccines. For example, Morishita et al. made use of exosomes derived from murine melanoma cells combined with adjuvant CpG to generate a cancer vaccine, successfully induced tumor antigen-specific immune response in mice [107]. EV originating from DC is another promising candidate for tumor vaccine. Antigen-loaded and matured DC-derived EVs with high expression of major histocompatibility complex and co-stimulatory molecules CD80 and CD86 are capable of directly stimulating antigen-reactive T cell responses [108, 109]. In murine tumor models, DC-derived EVs have been demonstrated to activate strong adaptive immune responses against cancers [110].

**Fig. 7:**
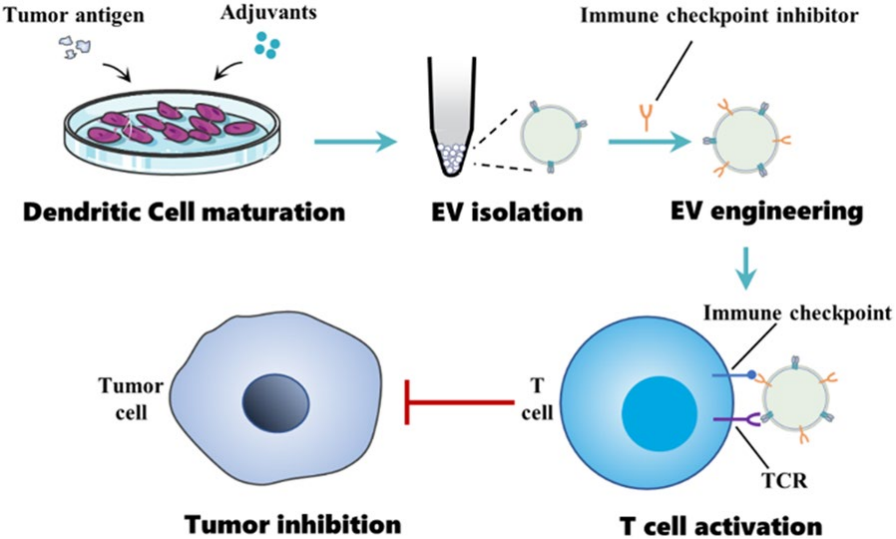
Engineered EVs for the combination of cancer vaccine with immune checkpoint blockade. EVs derived from mature dendritic cells are modified with immune checkpoint inhibitors. These engineered EVs can realize the combination of cancer vaccine and immune checkpoint blockade, effectively activating T cells to inhibit tumor growth

Although therapeutic tumor vaccines can induce anti-tumor immunity, they often failed to produce actual anti-tumor effects in many trials. With a better understanding of host immunity and TME, the immunosuppressive barriers in the TME has been proved to be a critical reason for the failure of cancer vaccines. “Cold” tumors have poor capacity to attract immune cell infiltration and immunosuppressive factors, which will weaken the functions of immune cells and make vaccine-induced immunity hard to form [111]. Combinational therapy provides a choice to overcome the problem, especially when tumor vaccines are combined with ICB therapy. Cancer vaccines promote anti-cancer immunity priming, and then ICI boost and maintain the vaccine’s effect to produce persistent immune responses. A series of studies have indicated the effectiveness of therapeutic cancer vaccine combined with immune checkpoint blockade therapy [112, 113]. Engineered EVs are also explored to apply in this combinational scheme (Fig. 7). In the Zhao et al. research, tumor-derived antigenic microparticles (T-MPs) were employed to load nano-Fe_3_O_4_ and then adjuvant CpG-loaded liposome arrays were bound to the surface of T-MPs to form the cancer vaccines (Fe_3_O_4_/T-MPs-CpG/Lipo). The combination of Fe_3_O_4_/T-MPs-CpG/Lipo vaccine and immune checkpoint PD-L1 blockade specifically inhibited ∼83% progression of B16F10-bearing mice [34]. In another typical study by Phung et al., exosomes derived from ovalbumin-pulsed, activated DCs were modified with anti-CTLA-4 antibody to generate bifunctional exosomes (EXO-OVA-mAb) combining vaccination and ICB. Their study showed that the functionalized EXO-OVA-mAb could synergize cancer vaccine efficacy and immune checkpoint inhibition to generate potent antitumor immune responses against the tumor [114]. These preliminary research results have shown that engineered EVs with inherent vaccine properties have great potential to realize the combination of a therapeutic cancer vaccine with ICB.

## Engineered EVs for visual therapy combining imaging and cancer treatment

**Fig. 8:**
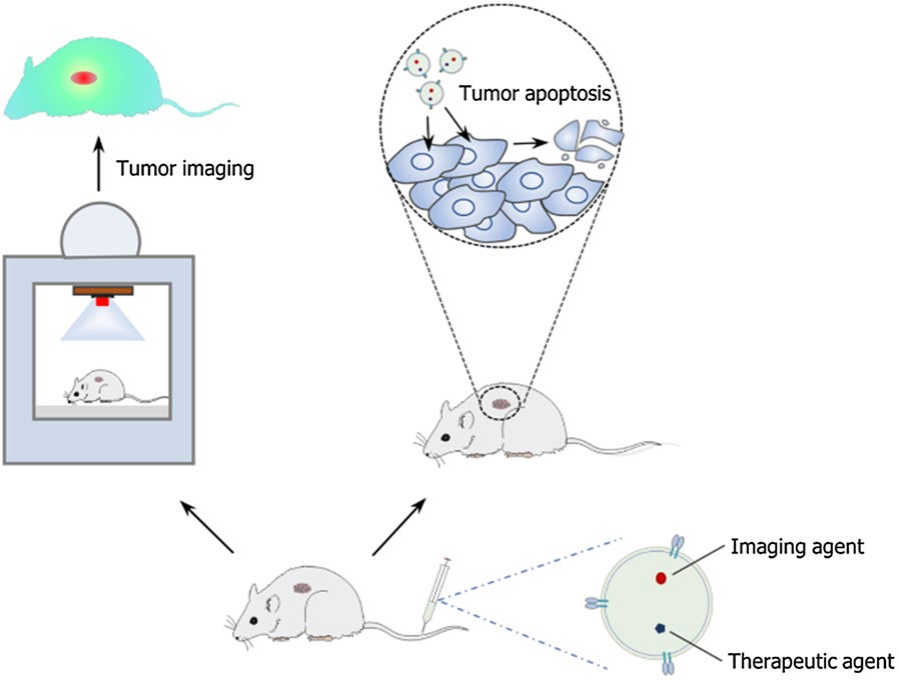
Engineered EVs for visual therapy. Co-delivery of imaging agent and therapeutic agent by engineered EVs can achieve both tumor imaging and treatment at the same time

Visual therapy is an ideal pattern of cancer therapeutics which can achieve both imaging and treatment simultaneously. It will significantly benefit the clinical work in the whole treatment process. Before the treatment, accurate imaging provides the location, size, and distribution of the tumor, determining whether it is suitable for therapy. In the treatment process, real-time imaging can track the distribution and metabolism of drugs, which can guide therapy precisely. After the treatment, curative effects can be monitored through the changes in the images of the lesion. Therefore, unexpected damage to normal tissues can be found and avoided promptly. Considering these strengths of visual therapy, researchers are committed to achieving the combination of cancer imaging and treatment. One of the most potent approaches to visual therapy is utilizing engineered EVs (Fig. 8). The load capacity and biocompatibility of engineered EVs are conducive to delivering imaging materials for multimodal imaging. Their precise targeting ability can further help label tumor cells and achieve targeted visual therapy. For example, in the glioma research of Jia et al. a new type of exosome for targeted imaging and therapy was designed. The engineered exosomes (RGE-Exo-SPION/Cur) were loaded with superparamagnetic iron oxide nanoparticles (SPION) and curcumin (Cur), and then their membrane was conjugated with neuropilin-1-targeted peptide (RGE) to obtain glioma-targeting ability. The study proved that RGE-Exo-SPION/Cur could penetrate the BBB smoothly and provided good results for targeted imaging, SPION-mediated magnetic flow hyperthermia therapy, and Cur-mediated therapy [32]. In another research by Fan et al. the strategy of using functionalized DNA to anchor quantum dots (QDs) onto EV surface was developed for tumor imaging and therapy. The labeling property of luminophores QDs endowed QD-engineered EVs with imaging function. When this strategy was applied to artificial vesicles of M1 macrophages loaded with DOX, it realized targeted label and treatment for visual therapy of tumor in mice [35]. Cao et al. also used the features of QDs to develop engineered exosomes for visual therapy. They first synthesized the vanadium carbide QDs modified with cell nucleus-target TAT peptides (V_2_C-TAT), then encapsulated the V_2_C-TAT QDs into exosomes engineered with cell target-RDG peptide. They formed a dual-target system of cell membrane and nucleus of cancer (V_2_C-PEG-TAT@Ex-RGD). Results showed that V_2_C-PEG-TAT@Ex-RGD could target the cell and enter the nucleus to accomplish low-temperature PTT and realized multimodal imaging, including fluorescent imaging, photoacoustic imaging, and magnetic resonance imaging [115]. All these prominent results suggest that engineered EVs are potential platforms for delivering imaging and therapeutic agents. Their noteworthy performance is helpful to achieve visual therapy for cancer precision treatment.

## Engineered EVs for other schemes of cancer combination therapy

Recently, researchers have developed some emerging cancer treatment methods and tried to combine them with traditional therapy to obtain better cancer treatment effects. Engineered EVs are also explored to apply in these schemes of combinational therapy. For example, carbon monoxide (CO), which can traverse tumor stroma and enter tumor cells to affect metabolism and energy production, has drawn growing concerns for cancer therapy [116]. The function of sensitizing other treatments such as radiotherapy makes CO have great potential to be combined with other cancer therapies. Zhu et al. used tumor-derived exosomes to deliver manganese carbonyl, which could react with H_2_O_2_ in the TME to produce CO, finally achieving the combination of cancer gas therapy and low dose radiotherapy to inhibit tumor growth in 4T1 mice [117].

Besides the various dual combinations of cancer treatment approaches mentioned above, some studies reported that engineered EVs could realize three or more combinational schemes in cancer treatment. For instance, Ding et al. indicated an engineered self-activatable photo-EV for synergistic trimodal anticancer therapy. In this study, M1 macrophage-derived EVs were prepared and simultaneously loaded with bis[2,4,5-trichloro-6-(pentyloxycarbonyl) phenyl] oxalate (CPPO), photosensitizer chlorin e6 (Ce6), and chemotherapy drugs prodrug aldoxorubicin (Dox-EMCH). M1 EVs with tumor-homing capability could target tumor sites and repolarize M2 to M1 macrophage to realize immunotherapy and produce H_2_O_2_. Then H_2_O_2_ would react with CPPO to generate chemical energy that activates Ce6, producing singlet oxygen (^1^O_2_) for PDT. Meanwhile, ^1^O_2_ brought up the rupture of EV membrane and induced the release of Dox-EMCH for chemotherapy. After injection into 4T1 tumor-bearing mice, these engineered EVs performed a perfect combination of immunotherapy, PDT, and chemotherapy in TME. It significantly suppressed tumor growth and prolonged the survival of tumor-bearing mice [118]. In another study by Wang et al., DOX was loaded into the exosomes coated with magnetic nanoparticles conjugated with molecular beacons. Under NIR radiation, these engineered exosomes could generate thermal energy to realize PTT and trigger cargoes release. The released molecular beacon could target the miR-21 for gene therapy, and DOX could perform chemotherapeutic effect. Synthetic chemo/gene/photothermal therapy of these engineered exosomes applied in tumor-bearing mice could reduce tumor size by 97.57% [119]. These innovative researches suggest that engineered EVs can display great diversity and achieve various combinational schemes in cancer treatment.

## Perspectives and challenges

The application of engineered EVs has emerged as a promising way to fight against cancer. Much progress has been made and the development trend mainly focused on combinational treatment in recent years. Therefore, deeper understanding of the EVs biology and better improvements in engineering strategies diminished the barrier for the application of engineered EVs to cancer combination therapy. A great number of studies have shown that engineered EVs with strong load capacity, high plasticity, good biocompatibility, and precise targeting capabilities are promising platforms to implement cancer combinational therapy, especially for the combinations related to chemotherapy or immunotherapy. Moreover, engineered EVs is very potential to further optimize the synthetic effect of the combination therapy to reach the desired therapeutic effect.

Nevertheless, some critical issues need to be resolved in this field. The first one is how to overcome the difficulty of mass production of engineered EVs. Up to now, it still costs much time to produce enough EVs for cancer treatment. The incomplete modification will further reduce the utilization ratio of EVs, which significantly limits the wide application in clinical trials. The second problem is the heterogeneity of EVs. The isolated EVs are very heterogeneous due to different sizes or contents. During the engineering process, different degrees of modification in each EV will lead to obvious heterogeneity. In addition, safety and targeted delivery are other issues that we should pay attention to when engineered EVs are applied in cancer combination therapy.

The issues above are particularly prominent in immune cell-derived EVs. Unlike tumor cells, human autologous immune cells are hard to obtain sufficient parent cells in vitro in short time. Coupled with the difficulty in mass production of EVs, it is laborious and costly to produce healing amounts of immune cell-derived EVs in practice. Moreover, primary human immune cells are difficult to isolate to 100% homogeneity, and their activation is often asynchronous and incomplete. Therefore, the obtained EVs from these immune cells often have significant heterogeneity, including immunostimulatory and tolerogenic EVs. These problems limit the effect of immune cell-derived EVs in this field.

Recently, some new technologies and methods have been developed to provide potential solutions to the above problems. For EVs mass production, there are two main development directions. One is to optimize the culture of source cells, and the other is to find more suitable stimulation to increase EV production per cell [120]. Multiple bioreactors like “flask” bioreactor and stirred tank bioreactors were designed for large-scale culture of source cells [121–123]. Mechanical stress was also proven and developed for the mass production of EVs [124]. As for the heterogeneity of EVs, single-vesicle and single-molecule analysis techniques to precisely analyze each EV and its cargo is a promising strategy. In addition, new advanced devices or techniques such as atomic force microscopy [125], super-resolution fluorescence microscopy [126], fluorescence correlation spectroscopy [127], nanoflow cytometry [128, 129], and single-particle interferometric reflectance imaging sensing (SP-IRIS) [130] were developed for obtaining more accurate characterization of each EV and its cargo [131]. For the EVs targeting ability, further revealing the mechanism of targeted delivery in EVs biology will be helpful to provide the theoretical basis for improving EV targeting capabilities [19].

Although most studies about engineered EVs and cancer combination therapy are still in the pre-clinical stage, technological advances and solutions to the problems mentioned above will shed light on the translation of engineered EVs from the bench to the bedside for cancer combination therapy in the future. More and better combinational schemes based on engineered EVs will also be developed to break the dilemma of cancer therapy.

## Conclusions

Engineered EVs have great potential in cancer combination therapy. In this review, we introduced current combinational schemes of engineered EVs and other cancer therapies. It is predictable that this area will be promising and more breakthroughs in cancer treatment will be achieved through more diverse combinational strategies of engineered EVs.

## Data Availability

Not applicable.

## Abbreviations

EV: Extracellular vesicle
TME: Tumor microenvironment
MDR: Multi-drug resistance
ICB: Immune checkpoint blockade
ILV: Intraluminal vesicle
TGN: Trans-Golgi network
MVE: Multivesicular endosome
BBB: Blood–brain barrier
P-gp: P-glycoprotein
DOX: Doxorubicin
miRNA: MicroRNA
5-FU: 5-fluorouracil
PTT: Photothermal therapy
PTA: Photothermal agents
ICG: Indocyanine green
NIR: Near-infrared
MSNs: Mesoporous silica nanoparticles
CRISPR: The clustered regularly interspersed short palindromic repeats
Cas9: CRISPR-associated proteins 9
PARP-1: Poly (ADP-ribose) polymerase-1
Bcl-2: B-cell lymphoma-2
NP: Nanoparticle
EPR: Enhanced permeability and retention
ROS: Reactive oxygen species
PTX: Paclitaxel
CuB: Cucurbitacin B
HSA: Human serum albumin
TAM: Tumor-associated macrophage
CAR: Chimeric antigen receptor
ICD: Immunogenic cell death
mPC: Metastatic peritoneal carcinoma
gETL NPs: Genetically engineered exosomes-thermosensitive liposomes hybrid nanoparticles
GM-CSF: Granulocyte–macrophage colony-stimulating factor
DTX: Docetaxel
PDAC: Pancreatic ductal adenocarcinoma
OXA: Oxaliplatin
PDT: Photodynamic therapy
PS: Photosensitizers
MSCs: Mesenchymal stem/stromal cells
ICI: Immune checkpoint inhibitors
DC: Dendritic cell
T-MPs: Tumor-derived antigenic microparticles
SPION: Superparamagnetic iron oxide nanoparticle
Cur: Curcumin
QDs: Quantum dots
CO: Carbon monoxide
Ce6: Chlorin e6
SP-IRIS: Single-particle interferometric reflectance imaging sensing
